# Regulation of Monocyte Adhesion and Migration by Nox4

**DOI:** 10.1371/journal.pone.0066964

**Published:** 2013-06-18

**Authors:** Chi Fung Lee, Sarah Ullevig, Hong Seok Kim, Reto Asmis

**Affiliations:** 1 Department of Biochemistry, School of Health Professions, University of Texas Health Science Center at San Antonio, Texas, United States of America; 2 Department of Clinical Laboratory Sciences, School of Health Professions, University of Texas Health Science Center at San Antonio, Texas, United States of America; University of Illinois at Chicago, United States of America

## Abstract

We showed that metabolic disorders promote thiol oxidative stress in monocytes, priming monocytes for accelerated chemokine-induced recruitment, and accumulation at sites of vascular injury and the progression of atherosclerosis. The aim of this study was to identify both the source of reactive oxygen species (ROS) responsible for thiol oxidation in primed and dysfunctional monocytes and the molecular mechanisms through which ROS accelerate the migration and recruitment of monocyte-derived macrophages. We found that Nox4, a recently identified NADPH oxidase in monocytes and macrophages, localized to focal adhesions and the actin cytoskeleton, and associated with phospho-FAK, paxillin, and actin, implicating Nox4 in the regulation of monocyte adhesion and migration. We also identified Nox4 as a new, metabolic stress-inducible source of ROS that controls actin *S*-glutathionylation and turnover in monocytes and macrophages, providing a novel mechanistic link between Nox4-derived H_2_O_2_ and monocyte adhesion and migration. Actin associated with Nox4 was *S*-glutathionylated, and Nox4 association with actin was enhanced in metabolically-stressed monocytes. Metabolic stress induced Nox4 and accelerated monocyte adhesion and chemotaxis in a Nox4-dependent mechanism. In conclusion, our data suggest that monocytic Nox4 is a central regulator of actin dynamics, and induction of Nox4 is the rate-limiting step in metabolic stress-induced monocyte priming and dysfunction associated with accelerated atherosclerosis and the progression of atherosclerotic plaques.

## Introduction

Atherosclerosis like other chronic inflammatory diseases is closely associated with systemic oxidative stress [1], [2]. The origins of the reactive oxygen species (ROS) that contribute to systemic oxidative stress are not clear since ROS can be generated by multiple different sources, including mitochondria or as by-products of numerous enzymatic reactions involving cytochrome P450 enzymes and 5-lipoxygenase, and resulting from the uncoupling of nitric oxide synthases [3]. However, at physiological levels, intracellular ROS are critical for cell signaling and regulate a large number of cellular functions, including cell proliferation, differentiation and motility as well cell survival [4], [5]. For cellular redox signaling to be specific and effective and to avoid non-specific oxidation, it is essential that ROS are generated in close proximity of their redox-sensitive targets [6], [7]. This is achieved by NADPH oxidases (Nox), professional ROS producers [8]. The original member of the Nox family is the phagocytic Nox gp91^phox^ or Nox2, which has been studied extensively for its role in the respiratory burst of neutrophils and macrophages [9], [10]. Since the discovery of Nox2, several additional Nox family members have been identified and shown to be involved in redox-sensitive signaling pathways [7], [11], [12], [13]. We identified Nox4 in monocytes and macrophages as a source of intracellular ROS involved in macrophage death [14]. We went on to show that Nox4 is differentially regulated during monocyte differentiation and induced by oxidized LDL. More recently, we showed that metabolic stress primes monocytes for activation by chemokines and that the transformation of monocytes into this hyper-responsive phenotype increases monocyte motility and requires the induction of Nox4 and increased H_2_O_2_ production [15].

The recruitment of monocyte-derived macrophages to sites of arterial injuries is a rate-limiting step of the initiation and progression of atherosclerosis [1]. Arterial injury triggers the expression of adhesion molecules production on the endothelium and promotes the synthesis and release of chemoattractants such as MCP-1 from the vascular wall. In response to these vascular signals, circulating monocytes adhere to the endothelium and transmigrate into the arterial wall. Previously, we showed that monocyte chemotaxis is accelerated by metabolic stress in a Nox4–dependent manner, but whether monocyte adhesion, the initiating step in monocyte transmigration, was also increased by metabolic priming was not known. In this study, we provided evidence that exposure to metabolic stress accelerates monocyte adhesion through a Nox4-dependent mechanism that does not require endothelial signals. Furthermore, we showed that accelerated adhesion facilitates and accelerates chemotaxis of primed monocytes. Monocyte priming appears to involve the recruitment of Nox4 to focal adhesions and the F-actin cytoskeleton, and the subsequent *S*-glutathionylation of actin.

## Materials and Methods

### Generation and Culture of Human Monocyte-derived Macrophages

Deidentified blood from healthy individuals was obtained from the South Texas Blood and Tissue Center. Mononuclear cells were isolated and separated by Ficoll gradient centrifugation and differentiated into mature human monocyte-derived macrophages (HMDM) in Teflon bag at 37°C for 14 days with 15% human serum (Valley Biomedical) as we described previously [16]. HMDM were plated on Aclars (ProPlastic) and washed repeatedly to remove non-adhering cells. The culture medium contained RPMI (Gibco) supplemented with 2 mM GLUTAMAX-1 (Gibco) 1% v/v non-essential amino acids (Gibco), 1 mM sodium pyruvate (Cellgro), 0.1 U/ml Penicillin G, 0.1 mg/ml streptomycin (Gibco), 10 mM HEPES (Gibco) as well as 5% human AB serum where indicated. The research involved human subjects but was determined to be EXEMPT by the UTHSCSA Review Board under DHHS Regulation 46.101 (b) category (4).

### THP-1 Monocyte Culture

THP-1 monocyte cell line was obtained from ATCC. The cells were cultured in RPMI medium supplemented with non-essential amino acids (Gibco), Glutamax-1 (Gibco), sodium pyruvate (Gibco), Penicillin-streptomycin (Gibco), and HEPES (Fluka). To mimic metabolic stress condition *in vitro*, the culture medium was supplemented with 20 mM D-glucose (final glucose concentration  = 25 mM) plus 100 µg/ml of freshly isolated human LDL (LDL+Glc). L-glucose instead of D-glucose was used for osmotic controls.

### LDL Isolation

LDL was isolated by NaBr gradient ultracentrifugation from pooled plasma obtained from healthy blood donors. LDL was purified by gel-filtration chromatography and filter-sterilized [17].

### Immunostaining and Confocal Microscopy

HMDM were fixed in 4% paraformaldehyde and permeabilized with 0.01% Triton X-100. BSA (1%) and donkey serum (4%) were used to for blocking. Macrophages were stained with rabbit Nox4 monoclonal antibodies [14], mouse paxillin (BD Transductions), rabbit Y397-phosphorylated FAK (Invitrogen), mouse COX IV (Abcam), mouse Lamp-1 (Abcam), mouse Golgin-97 (Invitrogen), and goat HSP60 antibodies (Santa Cruz). Alexa 488-labeled phalloidin and Alexa 488-labeled cholera toxin were from Invitrogen. Anti-mouse IgG Cy3 and anti-rabbit IgG Cy5 antibodies were from JacksonImmuno Research. Confocal images were collected from Olympus FV-1000 Laser Scanning Confocal Microscopy from Core Optical Imaging Facility from UTHSCSA. Degree of co-localization was analyzed and estimated with the intensity correlation analysis plug-in package of ImageJ.

### Immunoprecipitation

HMDM were lysed with lysis buffer (MES-buffered saline with 1% Triton X-100, 1 mM EDTA and 0.5% NP-40) containing protease inhibitor (Roche). Lysates were centrifuged at 15,000×g for 10 minutes, and supernatants were collected. The supernatant (approximately ∼10 mg of protein) was precleared with Protein G Sepharose (GE Healthcare) and then split into two aliquots of equal volume. Nox4 mAb or rabbit serum was added to the lysate for 4°C overnight with agitation. Each sample was centrifuged at 5000×g for 10 minutes to remove precipitated protein during overnight incubation. 10 μl pre-washed Protein G Sepharose was added to all samples, followed by overnight agitation at 4°C. Samples were then pelleted and washed with co-IP lysis buffer for three times. Samples were resuspended and heated in Laemmli buffer and subjected to Western blot analysis.

### Western Blot Analysis

Protein lysates was separated by SDS-PAGE, and proteins were then transferred to PVDF membranes (Millipore). Bands were detected by chemiluminescence (ECL chemilumnescence kit, Pierce) on a KODAK Image Station 4000 MM. Band intensities were and normalized to β-actin (Santa Cruz). Mouse p22^phox^ (Santa Cruz), rabbit Y397-phosphorylated FAK antibodies, mouse paxillin antibodies (BD Transductions), sheep LMW PTP (Exalpha Biological), mouse Lamp-1 (Abcam), mouse caveolin Y14Pi (BD Transductions), and mouse GSH antibodies (Millipore) were used according the manufacturers' recommendation.

### Intracellular ROS Assays

THP-1 monocytes were loaded for 2 h with 2′,7′-dichlorodihydrofluorescein diacetate (H_2_DCF-DA, Invitrogen) and then washed to remove excess dye. Labeled THP-1 monocytes were then primed with 100 µg/ml LDL and 25 mM glucose. Intracellular ROS formation was measured using a fluorescent plate reader as described previously [17], [18].

### siRNA Knockdown

Non-targeting siRNA (control) and siRNAs targeting human Nox4 were purchased from Dharmacon. siRNAs were transfected into THP-1 monocytes with GeneSilencer transfection reagent (Genlantis) for 24 h, followed by metabolic priming and subsequent ROS measurements or adhesion or chemotaxis assays.

### Adenoviruses

Adenoviruses expressing doxycycline-inducible Nox4 were generated using the pAdEasy system as described elsewhere [19]. Infections were performed at indicated MOIs for 12 h. Nox4 expression was initiated by the addition of doxycycline at indicated concentrations for 24 h.

### Monocyte Adhesion Assay

For each condition, 1×10^6^ THP-1 monocytes were plated for 45 minutes onto fibronectin-coated plates in either the absence or presence of 2 nM MCP-1, and then washed with PBS to remove non-adhering cells. To quantify cell adhesion, adhered monocytes were collected, and cell numbers were determined using the PicoGreen® ds DNA quantitation assay (Invitrogen).

### Monocyte Chemotaxis Assay

THP-1 monocytes were then loaded into the upper wells of a 48-well modified Boyden chamber (NeuroProbe). The lower wells contained 2 nM MCP-1 (R&D Systems). A 5 µm polyvinyl pyrrolidone-free polycarbonate filter membrane was layered between the two sides of the chamber, and the chambers were incubated for 3 h at 37°C and 5% CO_2_.The membrane was washed gently and transmigrated cells from the upper side of the filter were removed. Transmigrated cells were stained and visualized with Diff-Quik^®^ Set (Dade Behring). Five separate high power fields were used to calculate cell migration under a light microscope (400×).

### Statistical Analysis

Results are expressed as mean ± standard deviation (SD) of at least 3 independent experiments. Data were analyzed by paired two-tailed *t* test for comparison between two groups. For multiple groups, we used one-way ANOVA test to compare values among all groups, and Student Newman-Keuls test for pairwise comparisons. GraphPad Prism 5 was used for the statistical analyses. A *P*-value of less than 0.05 was considered statistically significant.

## Results

### Nox4 localizes to actin and focal adhesions

**Figure 1:**
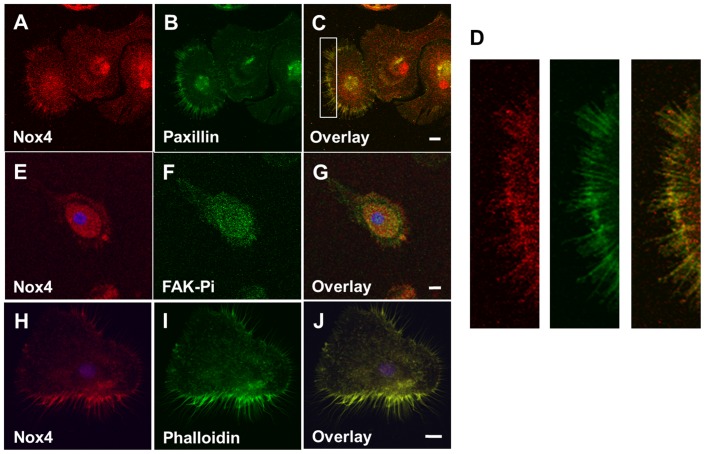
Nox4 localizes to focal adhesions and the F-actin cytoskeleton in mature human monocyte-derived macrophages. HMDM were fixed, permeabilized and stained as described in “Materials and Methods.” Nuclei were also stained by DAPI (blue). **A–C: Nox4 co-localizes with paxillin.** HMDM were labeled with antibodies directed against Nox4 (red) and paxillin (green). Colocalization is indicated in yellow (Pearson coefficient  = 0.79). **D**: Magnification of filed highlighted in panel C with white box in C showing the red (Nox4) and green signals (paxillin) and the overlay. **E–G: Nox4 co-localizes with activated FAK.** HMDM were labeled with antibodies directed against Nox4 (red) and FAK-Y397Pi (green). Colocalization is indicated in yellow (Pearson coefficient  = 0.75). **H–J: Nox4 co-localizes with F-actin**. HMDM were labeled with antibodies directed against Nox4 (red) and stained with Alexa Fluor 488-labeled phalloidin (green). Colocalization is indicated in yellow (Pearson coefficient  = 0.84). Scale bar  = 10 µm.

To elucidate the functional roles of monocytic Nox4, we first examined the sub-cellular localization of endogenous Nox4 protein. In vascular smooth muscle cells Nox4 was shown to co-localize with vinculin within focal adhesions, implicating Nox4 in cell adhesion and migration [20]. To examine whether Nox4 is present in focal adhesions of macrophages, we performed immunofluorescence studies in adherent HMDM stained with antibodies directed against paxillin, a focal adhesion protein that serves as a docking protein for cytoskeletal protein and kinases, including focal adhesion kinase (FAK) [21], and against the activated form of FAK, i.e. FAK phosphorylated at tyrosine-397 (FAK-Y397Pi) [22], a marker of activated focal adhesions. We observed extensive colocalization of Nox4 with paxillin throughout HMDM (Fig. 1A–C), but the colocalization was particularly pronounced in plasma membrane protrusions (Fig. 1D). Nox4 immunostaining also showed significant co-localization of Nox4 with FAK-Y397Pi (Fig. 1E–G), further linking Nox4 to the formation of the focal adhesion complex.

The formation of membrane protrusions and focal adhesions is essential for cell adhesion and motility and requires the dynamic turnover of actin [23]. In human macrophages we observed significant co-localization of Nox4 with phalloidin staining, suggesting that Nox4 is in close proximity of or directly associated with the F-actin cytoskeleton (Fig. 1H–J). Again, the colocalization was particularly pronounced in membrane protrusions, further supporting a role for of Nox4 in cell adhesion and migration. We also examined whether Nox4 localizes to other organelles or membranes within the cells. We did not observe any significant co-localization of Nox4 with Golgin-97, Lamp-1 or cholera toxin, a marker for lipid rafts (Fig. S1A–C), indicating that Nox4 is not present in the Golgi apparatus, lysosomes or the plasma membrane of macrophages.

In the heart, Nox4-derived ROS have been implicated in the regulation of mitochondrial ROS generation [24] and Nox4 has been reported to localize to mitochondria of cardiac myocytes [25]. However, in human macrophages, we did not observe any significant co-localization of Nox4 with either COX IV or HSP60 (Fig. S1D+E), two mitochondrial markers, suggesting that in macrophages Nox4 may not be localized at or associates with mitochondria. Several reports describe a role for Nox4 in redox signaling in the nucleus [26], [27]. Using biochemical fractionation approaches, we previously identified Nox4 in the nuclear fraction of HMDM [14]. Here, we now confirmed these findings by immunofluorescence and confocal microscopy studies (Fig. S1F), suggesting that Nox4 may also plays a role in the nuclear redox signaling of macrophages.

**Figure 2:**
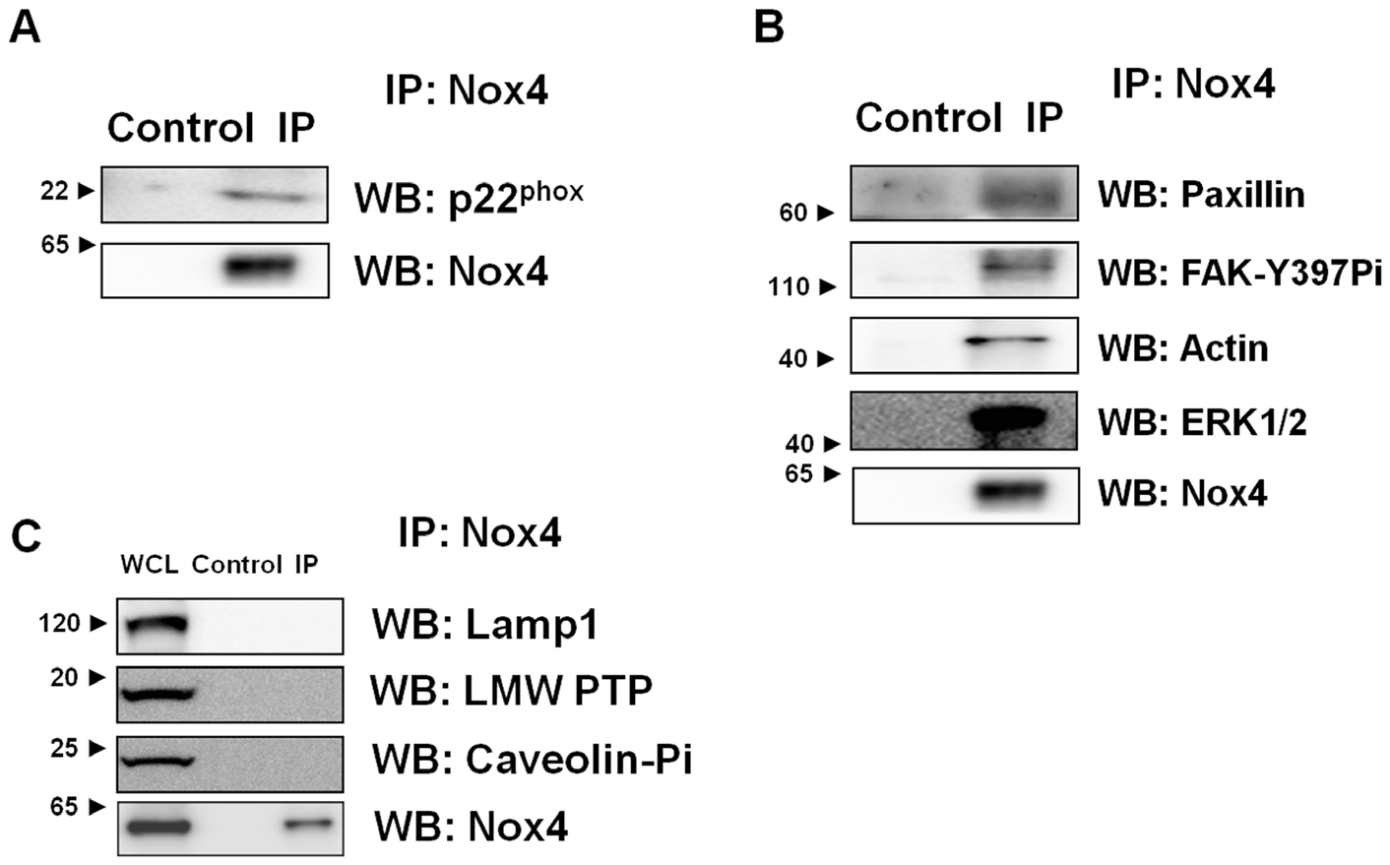
Nox4 co-immunoprecipitates with components of focal adhesions. Lysates of HMDM were treated with either rabbit monoclonal antibodies directed against Nox4 (IP) or rabbit serum (Control), and antibodies were precipitated with pre-washed Protein G Sepharose as described under “Materials and Methods.” Immunoprecipitates were separated by SDS-PAGE and blots were probed for p22^phox^ (**A**), known components of focal adhesions (**B**) and proteins presumably unrelated to focal adhesions (**C**). Representative images of at least 3 independent experiments are shown. WCL: whole cell lysate.

To further support a direct association of Nox4 with the cell adhesion machinery (Fig. 1), we conducted co-immunoprecipitation studies using our rabbit monoclonal Nox4 antibody [14]. As a positive control, we first confirmed in the Nox4 immunoprecipitate the presence of p22^phox^, the obligate dimerization partner of Nox4, (Fig. 2A). Consistent with our confocal microscopy findings, Nox4 also co-immunoprecipitated with paxillin, active FAK (FAK-Y397Pi) and actin (Fig. 2B). Interestingly, we also detected the MAPK ERK1/2, which was shown to be recruited to newly formed adhesion sites [28], where, by phosphorylating calpain, ERK1/2 regulates adhesion turnover [29].

As predicted from our confocal microscopy studies (Fig. S1B), we failed to detect the lysosomal marker Lamp1 in Nox4 immunoprecipitates (Fig. 2C). Interestingly, we also did not detect other signaling molecules reported to either be involved in redox-sensitive events, such as the redox-sensitive low molecular weight protein tyrosine phosphatase (LMW-PTP) [30], or in the reorganization of the plasma membrane, such as phosphorylated caveolin, a component of focal adhesions involved in lipid raft internalization [31] (Fig. 2C). These data suggest a level of selectivity and specificity for the interactions of Nox4 with components of focal adhesions. Collectively, our data support the concept that in monocytic Nox4 is directly involved in the formation and turnover of focal adhesions, further implicating Nox4 in the regulation of monocyte and macrophages adhesion.

### Increased Nox4 expression and ROS production is associated with enhanced monocyte adhesion

**Figure 3:**
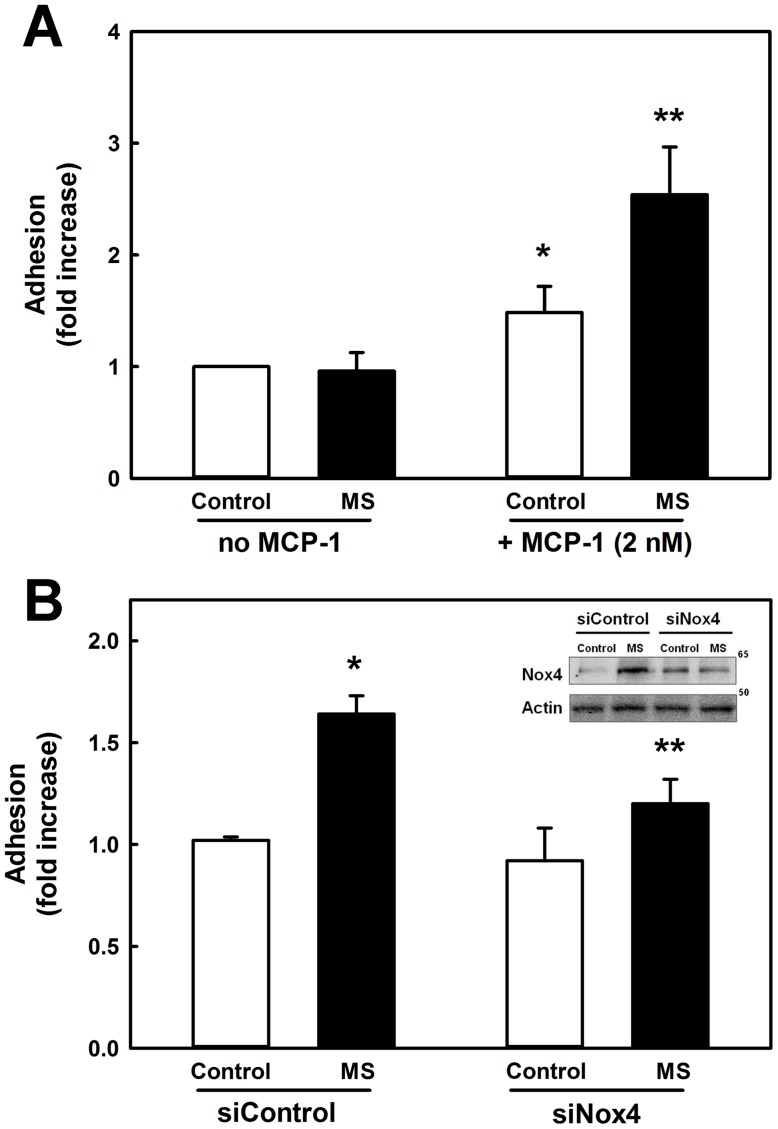
Nox4 knockdown prevents increased monocyte adhesion induced by metabolic stress. (**A**) THP-1 monocytes were either treated for 24 h with control medium (5 mM glucose, 2% FBS, open bars) or metabolically primed in control medium supplemented with 100 μg/ml native LDL plus 20 mM D-glucose (final concentration 25 mM, **MS**, closed bars). Cells were then allowed to adhere to fibronectin-coated wells either in the presence or absence of MCP-1 (2 nM) as described in “Materials and Methods.” *: *P*<0.05 vs. “Control – no MCP-1”; **: *P*<0.05 vs. “Control + no MCP-1,” n = 4. (**B**) THP-1 monocytes were transfected for 24 h with either scrambled (siControl) or Nox4-targetting siRNA (siNox4), and subsequently metabolically primed for 24 h where indicated (solid bars). Monocyte adhesion in response to MCP-1 (2 nM) was measured as described in “Materials and Methods”. **Insert:** Representative Western blot showing suppression of Nox4 protein levels by siRNA. *: *P*<0.05 vs. Control. **: *P*<0.05 vs. MS (siControl), n = 4.

Previously, we showed that chronic exposure of monocytes to diabetic conditions induced by human low-density lipoproteins plus high concentrations of D-glucose (LDL+HG) induced Nox4 expression, increased intracellular H_2_O_2_ formation, and primed monocytes for accelerated chemotaxis in response to chemokines [32]. We therefore explored whether metabolic stress-induced priming also increases monocyte adhesion. We found that the same conditions of metabolic stress, which induce Nox4 expression (Fig. S2A+B) and increase intracellular ROS formation (Fig. S2C), also increased 1.7-fold MCP-1-induced monocyte adhesion to fibronectin (Fig. 3A). Interestingly, in the absence of cytokine activation, metabolic stress did not affect adhesion of THP-1 monocytes to fibronectin. To confirm that increased adhesion of metabolically primed monocytes also resulted in accelerated migration, THP-1 cells from the same experiment were also subjected to the chemotaxis assay. Indeed, as expected from our previously reported studies, monocyte chemotaxis was increased 2.7-fold in metabolically primed monocytes (Fig. S2D). Nox4 induction only occurred in the presence of D-glucose but not L-glucose, and only D-glucose, not L-glucose, increased the priming effect of LDL, demonstrating that the priming effect of LDL+HG was not due to osmotic effects (Fig. S3).

### Nox4 knockdown suppresses metabolic stress-enhanced monocyte adhesion and chemotaxis

Next, we determined whether Nox4 induction was required for enhanced adhesion by metabolically-primed monocytes. Using lipid-mediated transfection, we introduced into THP-1 monocytes siRNAs with either a non-targeting sequence (siControl) or a sequence targeting human Nox4 mRNA (siNox4). Knockdown by siNox4 suppressed metabolic stress-induced Nox4 expression by 77% (Fig. 3B insert, Fig. S4A), and reduced by 85% ROS production stimulated by metabolic stress (Fig. S4B). Importantly, metabolically primed monocytes, in which Nox4 was knocked down, showed a 62% reduction in MCP-1-stimulated adhesion (Fig. 3B). The same cells also showed a 60% reduction in chemotaxis (Fig. S4C), confirming similar findings we reported previously ^1^.

### Adenovirus-mediated Nox4 over-expression promotes monocyte adhesion and chemotaxis

**Figure 4:**
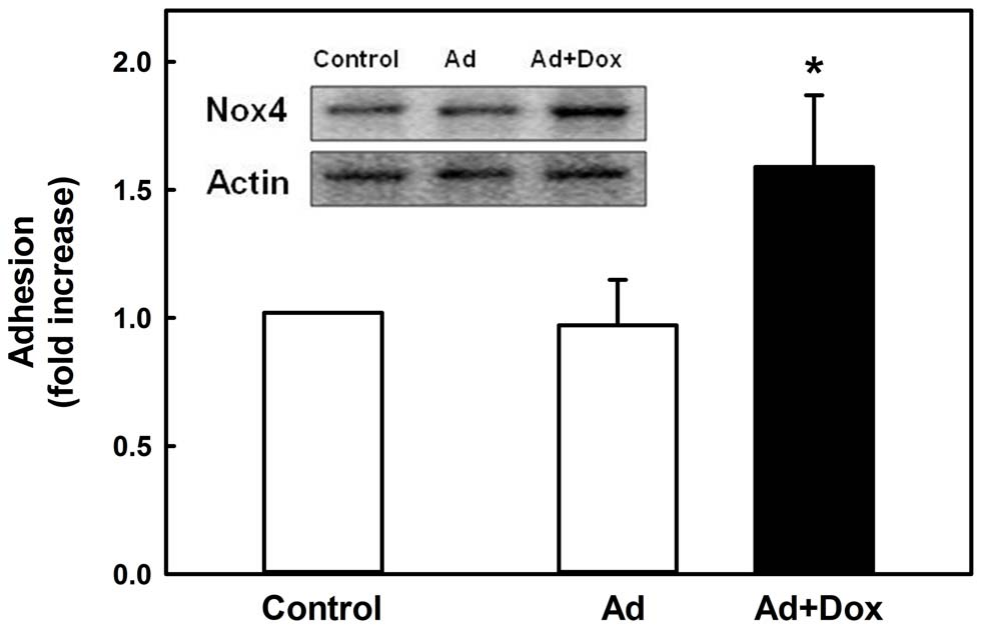
Overexpression of Nox4 mimics metabolic priming and promotes monocyte adhesion. THP-1 monocytes were infected with adenoviruses (Ad, MOI = 50) expressing Nox4 under the control of a doxycycline (Dox)-sensitive promoter. Nox4 expression was induced by adding Dox (1 ug/ml) to the culture medium. Monocyte adhesion in response to MCP-1 (2 nM) was measured as described in “Materials and Methods”. **Insert:** Representative Western blot showing adenovirus-mediated overexpression of Nox4. *: *P*<0.05 vs. “Ad”; n = 4.

To demonstrate that induction of Nox4 is sufficient to prime monocytes for the exaggerated adhesive response observed in metabolically stressed monocytes, we infected THP-1 monocytes with adenoviruses carrying the human Nox4 gene under the control of a doxycycline-inducible promoter [14]. Compared to uninfected control cells, adenovirus-infected THP-1 monocytes showed no change in their rate to adhere to fibronectin. However, when we induced Nox4 expression with doxycycline and increased Nox4 protein levels by 66% over endogenous Nox4 levels (Fig. 4 insert, Fig. S5A), monocyte adhesion was increased 1.6-fold (Fig. 4), mirroring the effects of metabolic priming. The increase in Nox4 was paralleled by an 89% increase in intracellular ROS production (Fig. S5B), confirming that the transgene was active. In agreement with our previous report [32], the same Nox4-overexpressing cells also showed a 2-fold increase in chemotactic activity (Fig. S5C), demonstrating that increased adhesion contributes to the overall increase in motility observed in metabolically primed monocytes. Collectively, these results support a central and essential role for Nox4 in mediating the effects of metabolic priming on monocyte adhesion and chemotaxis.

### Nox4 mediates actin-*S*-glutathionylation induced by metabolic stress

**Figure 5:**
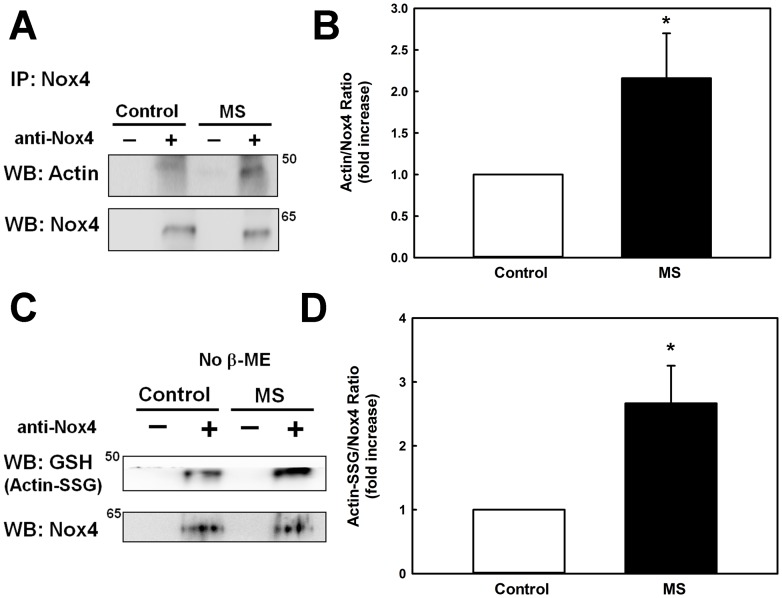
Nox4 association with actin and S-glutathionylation of Nox4-associated actin are increased metabolically stressed monocytes. THP-1 monocytes were either treated for 24 h with control medium (5 mM glucose, 2% FBS, Control, open bars) or metabolically primed in control medium supplemented with 100 μg/ml native LDL plus 20 mM D-glucose (final concentration 25 mM, **MS**, closed bars). Lysates of control cells and metabolically primed THP-1 monocytes were prepared and subjected to immunoprecipitations using rabbit monoclonal antibodies directed against Nox4 (+) or rabbit serum as a control (**−**). The lysates were pre-cleared by protein G sepharose and co-immunoprecipitation experiments were performed as detailed in “Materials and Methods.” When the co-IP experiments were repeated with protein G beads only (no serum), no actin was observed in the pull down. To assess the association of Nox4 with actin, Western blots were probed with antibodies directed against Nox4 and actin (**A+B**). To determine whether actin associated with Nox4 was *S*-glutathionylated, protein separation was conducted under non-reducing conditions, and Western blots were probed with antibodies directed against Nox4 and glutathione (GSH), the latter to identify *S*-glutathionylated actin (Actin-SSG; **C+D**). Panels B and D show the densitometric analysis of gels from three independent experiments. *:P<0.05 vs. Control; n = 3.

Previously, we reported that in monocytes metabolic stress promotes the *S*-glutathionylation of actin [32], a modification known to prevent the polymerization of G-actin [33]. We went on to show that Nox4 appears to be required for both actin-*S*-glutathionylation and increased actin turnover, but the mechanism underlying this reversible modification of actin had not been identified. Our co-immunoprecipitation and confocal microscopy studies (Fig. 1J and 2B) now suggest a direct association of Nox4 with the F-actin cytoskeleton. We therefore hypothesized that the association of Nox4 with actin promotes actin *S*-glutathionylation. To test this hypothesis, we used our Nox4 antibody to conduct immunoprecipitation experiments in lysates from untreated and metabolically primed monocytes. These pulldown experiments revealed that in metabolically primed monocytes 2.2-fold more actin precipitated with Nox4 than in unprimed cells (Fig. 5A+B), indicating an increased association of Nox4 with actin. To examine whether the association of actin with Nox4 promotes actin *S*-glutathionylation, the immunoprecipitate was separated by SDS-PAGE under non-reducing conditions and the membrane was probed with anti-GSH antibody. We detected a strong band with the same molecular weight as actin (Fig. 5C). When we treated the immunoprecipitate with β-mercaptoethanol prior to Western blot analysis, the anti-GSH antibody failed to detect any bands, suggesting this band was in fact *S*-glutathionylated actin (not shown). In immunoprecipitates from metabolically primed monocytes the intensity of the putative *S*-glutathionylated actin band increased 2.6-fold when normalized to Nox4 (Fig. 5D), i.e. an increase very similar to that observed for total actin (Fig. 5B). Together, our data strongly suggest Nox4 binds to actin and that this association promotes the *S*-glutathionylation of actin.

## Discussion

Monocyte recruitment is a central mechanism in immune defense and wound healing. This process is initiated by chemokine-induced contact of the monocyte with either the activated endothelium or the matrix of the injured vessel, resulting in monocyte adhesion. Monocyte adhesion is a prerequisite for monocyte emigration from the blood and migration to sites of injury or infection. However, monocyte recruitment is also involved in chronic inflammatory diseases and is the rate limiting step for the development atherosclerosis. In fact, recent evidence suggests that metabolic disorders not only initiate atherogenesis by promoting vascular injury, but dyslipidemia and hyperglycemia may directly convert monocytes into a proinflammatory, proatherogenic phenotype [34], [35]. However, the molecular mechanisms underlying monocyte dysfunction have remained obscured. We recently showed that metabolic stress “primes” monocytes for enhanced responsiveness to chemokines, increasing chemotaxis and monocyte recruitment, and that this process was mediated by Nox4 [32], [36]. The goals of the present study were to provide insights into the mechanism of monocyte priming, to determine how metabolic priming affects monocyte adhesion and chemotaxis, and to decipher the role for Nox4 in these processes. Here we show that (1) Nox4 is the source of ROS in monocytes that mediate monocyte priming induced by metabolic stress; (2) Nox4 is both necessary and sufficient to accelerate adhesion and chemotaxis of monocytes; (3) Nox4 is recruited to focal adhesions and (4) associates with the F-actin cytoskeleton, where it promotes actin-S-glutathionylation.

Adhesion and actin dynamics are crucial in regulating cellular motility [37]. The assembly of focal adhesion requires actin polymerization, and conversely, nucleations provided by adhesion support actin polymerization [38]. Actin fibers direct local protrusions through polymerization and drive the movement of adhesion molecules [39]. A tight correlation between adhesion assembly and actin polymerization supports the notion that interactions of adhesion components with actin fibers mediate new adhesions and thus migration [40]. In addition, direct interactions of FAK with Arp2/3 were observed [41], [42], suggesting the coupling of the two processes. Our data now suggest that in human macrophages these processes are redox-regulated and point to Nox4 as the source of ROS regulating both the adhesion and actin dynamics. Our confocal imaging and immunoprecipitation studies show that Nox4 localizes to focal adhesions (paxillin and phospho-FAK) and associates with the actin cytoskeleton. Although it is well-established that ROS play important roles in cell migration by regulating focal adhesions [20], [43], [44], [45] and the cytoskeleton [33], [46], [47], a variety of different sources for these ROS have been proposed. For example, 5-lipoxygenase and mitochondria are proposed to be the major source of ROS responsible for fibronectin/integrin-mediated adhesion, a process reported in fibroblasts to be regulated through the oxidative modification of low molecular weight protein tyrosine phosphatase (LMW-PTP) and the dissociation of LMW-PTP from FAK [43], [45], [48]. In endothelial cells, Nox2 localizes to sites of lamellipodial focal adhesions [49], while in smooth muscle cells Nox4 co-localizes with vinculin in focal adhesions [20], suggesting that NADPH oxidases may play a more general role in regulating cell adhesion. A recent study in macrophages isolated from Nox2-deficent mice suggested that Nox2 may be required for monocyte chemotaxis toward MCSF, and that Nox2 may provide a physiological ‘tone’ of ROS for monocytes and macrophages to sustain key functions [50]. However, the transfer of Nox2-deficient bone marrow into apoE^−/−^ mice had no significant effect on the extent of atherosclerotic plaques in 24-week old animals despite a profound reduction in basal and PMA-induced superoxide formation in Nox2^−/−^ macrophages [51]. Thus, these latter findings would suggest that Nox2 is not essential for monocyte recruitment *in vivo*. We recently showed that knockdown of Nox4 protected monocytes from metabolic priming without affecting Nox2 levels, also ruling out a significant role for Nox2 in metabolic stress-induced monocyte dysfunction [32]. Our study here further supports this role for Nox4 as we were able to show that in human monocytes the rate of cell adhesion and migration was tightly coupled with the expression level of Nox4. Overexpression of Nox4 accelerated both monocyte adhesion and chemotaxis, mimicking the effects of metabolic priming in these cells, whereas knockdown of Nox4 protected monocytes from metabolic stress-induced dysfunction. Our data suggest that Nox4-derived ROS play an important physiological role in orchestrating and regulating monocyte migration and thus macrophage recruitment.

Redox regulation of proteins is achieved by reversible oxidative modification of ionizable cysteine thiols, which are reactive with hydrogen peroxide (H_2_O_2_), but much less so with superoxide [52], [53]. Several lines of evidence suggest that Nox4 primarily generates H_2_O_2_ [54], [55], [56] and delivers ROS in a specific manner by localizing to its targets [6], [57], [58], strongly supporting a role for Nox4 in redox signaling. Actin is one of the better-known redox-sensitive proteins [33], [46]. Actin dynamics, and thus cell migration, are regulated in part by reversible *S*-glutathionylation, the formation of mixed disulfides between protein thiols and glutathione. Wang et al. first demonstrated that the reversible *S*-glutathionylation of actin at Cys374 promotes actin depolymerization [33]. Chiarugi and co-workers showed that preventing the oxidative modification of actin by mutating Cys374 to alanine inhibits cell spreading [46]. Nox4 co-localizes with α-actin in smooth muscle cells [58] and is found in actin-rich invadopodia of cancer cells [59], suggesting that Nox might be involved in the *S*-glutathionylation and redox regulation of actin. Our data now provide compelling evidence that Nox4 regulates monocyte adhesion and migration via oxidative modification and *S*-glutathionylation of actin. We show that metabolic stress promotes the association of Nox4 with actin, and that this association results in increased levels of actin S-glutathionylation. Recruitment of Nox4 to actin allows Nox4 to generate ROS in close proximity to actin and thus selectively oxidize actin, presumably by oxidizing a cysteine thiols to sulfenic acids [60], which after forming a mixed disulfide with glutathione, yields *S*-glutathionylated actin. The enhanced actin-*S*-glutathionylation in monocytes in response to metabolic stress is consistent with the increase in thiol oxidative stress we observed in peritoneal macrophages isolated from both dyslipidemic and diabetic mice [15]. Continuous assembly and disassembly of the actin cytoskeleton and focal adhesions are required for the increase in rate of migration. We recently showed that even under resting conditions, a fraction of actin is *S*-glutathionylated, dramatically reducing the ability of G-actin to polymerize into F-actin [32]. In response to physiological stimuli such as EGF, actin is de-glutathionylated, resulting in an increased rate of polymerization and F-actin formation. In this study, Nox4 appears to localize at the F-actin cytoskeleton. One potential explanation could be that Nox4 promotes the dynamic disassembly of actin by S-glutathionylating F-actin, thereby accelerating F-actin depolymerization and actin turnover. This process appears to be counter-regulated by glutaredoxin, analogous to the deglutathionylation of actin in response to EGF [32]. However, Nox4 localization in human macrophages was not limited to the actin cytoskeleton. It is possible that Nox4-derived ROS may act on other redox-sensitive proteins involved in actin turnover and cell adhesion, such as FAK and PTP, and that Nox4 may regulate actin dynamics and monocyte adhesion and migration through a more complex and comprehensive mechanism. While the molecular events underlying Nox4 translocation within the monocytes and macrophages and the mechanisms that allow Nox4-derived ROS to selectivity modify target proteins are not yet fully understood, our studies have now identified Nox4-dependent *S*-glutathionylation of actin as a novel mechanism regulating monocyte adhesion and migration.

In summary, our study provides new insights into the role of Nox4 in monocyte adhesion and migration and new molecular details of the mechanisms involved in monocyte dysfunction associated with metabolic disorders and chronic inflammatory diseases, including atherosclerosis.

## Supporting Information

Figure S1 **Sub-cellular localizations of Nox4.** (**A**) HMDM were immunostained with Nox4 (red) and Golgin-97 antibodies (green). Pearson coefficient  = 0.278. (**B**) HMDM were stained with Nox4 (red) and Lamp-1 antibodies (green). Pearson coefficient  = 0.167. (**C**) HMDM were stained with Nox4 (red) and Cholera toxin (green). (**D**) HMDM were labeled with Nox4 (red) and COX IV antibodies (green). Pearson coefficient  = 0.323. (**E**) HMDM was immunostained with Nox4 (red) and HSP60 antibodies (green). Pearson coefficient  = 0.649. (**F**) HMDM were stained with Nox4 antibody and DAPI. Z-sectioning images showed Nox4 staining in the nucleus. Bar represents 10 µm.
(TIF)

Figure S2 **Metabolic stress concomitantly increases Nox4 protein levels, ROS formation and monocyte chemotaxis.** (**A**) Nox4 protein levels were measured by Western blot analysis in control or metabolically primed (MS) THP-1 monocytes. (**B**) Quantification of Nox4 protein levels. (**C**) ROS formation in DCFH-loaded control or metabolically primed (MS) THP-1 monocytes as described under “Material and Methods.” (**D**) Monocyte chemotaxis in response to MCP-1 was measured in control or metabolically primed (MS) THP-1 monocytes. *: *P*<0.05 vs control.
(TIF)

Figure S3 **Only D-glucose, not L-glucose, induces Nox4 expression and accelerates monocyte chemotaxis.** (**A**) THP-1 monocytes were preincubated with either 20 mM L-glucose plus 5 mM D-glucose or 25 mM D-glucose where indicated and Nox4 expression was determined. Representative Western blots are shown. (**B**) Quantification of Nox4 expression levels. n = 2.
(TIF)

Figure S4 **Knockdown of Nox4 suppressed metabolic stress-induced ROS formation and accelerated chemotaxis.** THP-1 monocytes were transfected with control or Nox4 siRNAs and then stimulated with control or metabolic stress medium. (**A**) Nox4 protein expression, (**B**) ROS formation and (**C**) MCP-1-induced chemotaxis were measured as described under “Material and Methods.” **: *P*<0.05 vs MS/siControl. *: *P*<0.05 vs C/siControl; n = 4.
(TIF)

Figure S5 **Overexpression of Nox4 promotes OS production and accelerates monocyte chemotaxis.** (**A**) Nox4 was overexpressed in THP-1 monocytes using doxycycline-inducible adenoviruses (MOI = 50; 1 μg/ml Dox). (**B**) ROS production and (**C**) chemotaxis was measured as described under “Material and Methods”. *:*P*<0.05 versus Control; n = 4.
(TIF)
