# Supplementary figures and images for: Regulation of Monocyte Adhesion and Migration by Nox4
Source: PLoS One. 2013 Jun 18;8(6):e66964. doi: 10.1371/journal.pone.0066964 (PMC3688996; doi:10.1371/journal.pone.0066964)

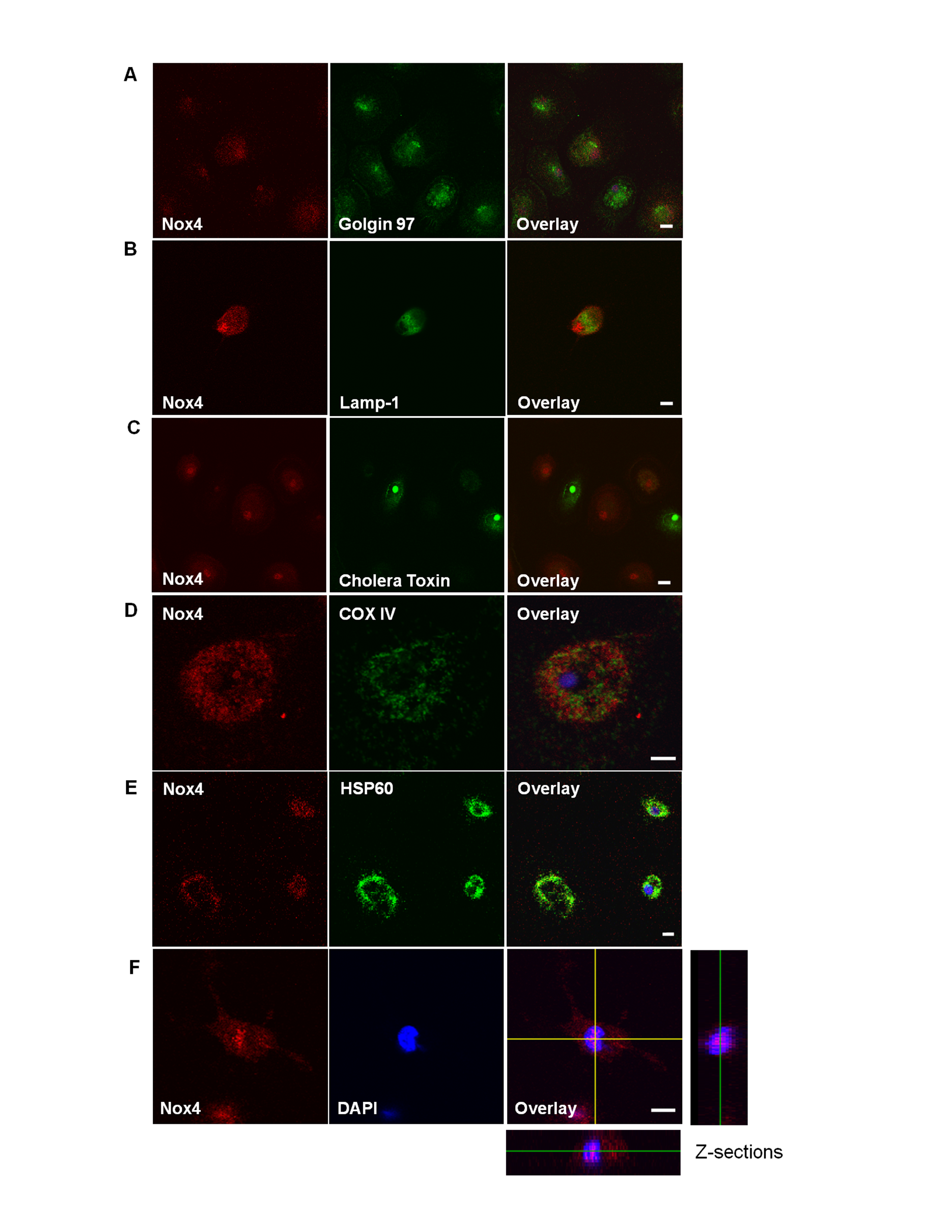

Supplement: Figure S1 — Sub-cellular localizations of Nox4. (A) HMDM were immunostained with Nox4 (red) and Golgin-97 antibodies (green). Pearson coefficient = 0.278. (B) HMDM were stained with Nox4 (red) and Lamp-1 antibodies (green). Pearson coefficient = 0.167. (C) HMDM were stained with Nox4 (red) and Cholera toxin (green). (D) HMDM were labeled with Nox4 (red) and COX IV antibodies (green). Pearson coefficient = 0.323. (E) HMDM was immunostained with Nox4 (red) and HSP60 antibodies (green). Pearson coefficient = 0.649. (F) HMDM were stained with Nox4 antibody and DAPI. Z-sectioning images showed Nox4 staining in the nucleus. Bar represents 10 µm. (TIF) [file pone.0066964.s001.tif]

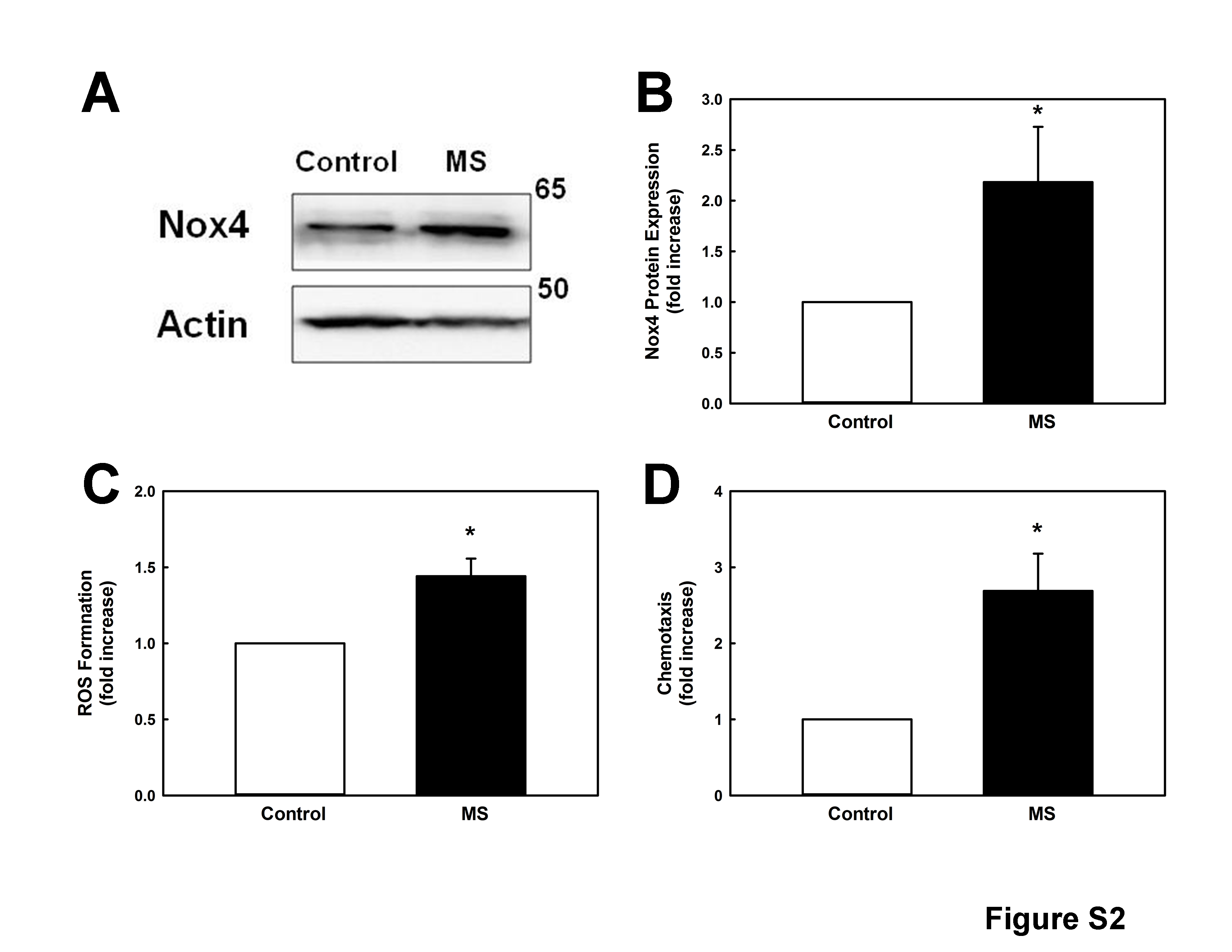

Supplement: Figure S2 — Metabolic stress concomitantly increases Nox4 protein levels, ROS formation and monocyte chemotaxis. (A) Nox4 protein levels were measured by Western blot analysis in control or metabolically primed (MS) THP-1 monocytes. (B) Quantification of Nox4 protein levels. (C) ROS formation in DCFH-loaded control or metabolically primed (MS) THP-1 monocytes as described under “Material and Methods.” (D) Monocyte chemotaxis in response to MCP-1 was measured in control or metabolically primed (MS) THP-1 monocytes. *: P<0.05 vs control. (TIF) [file pone.0066964.s002.tif]

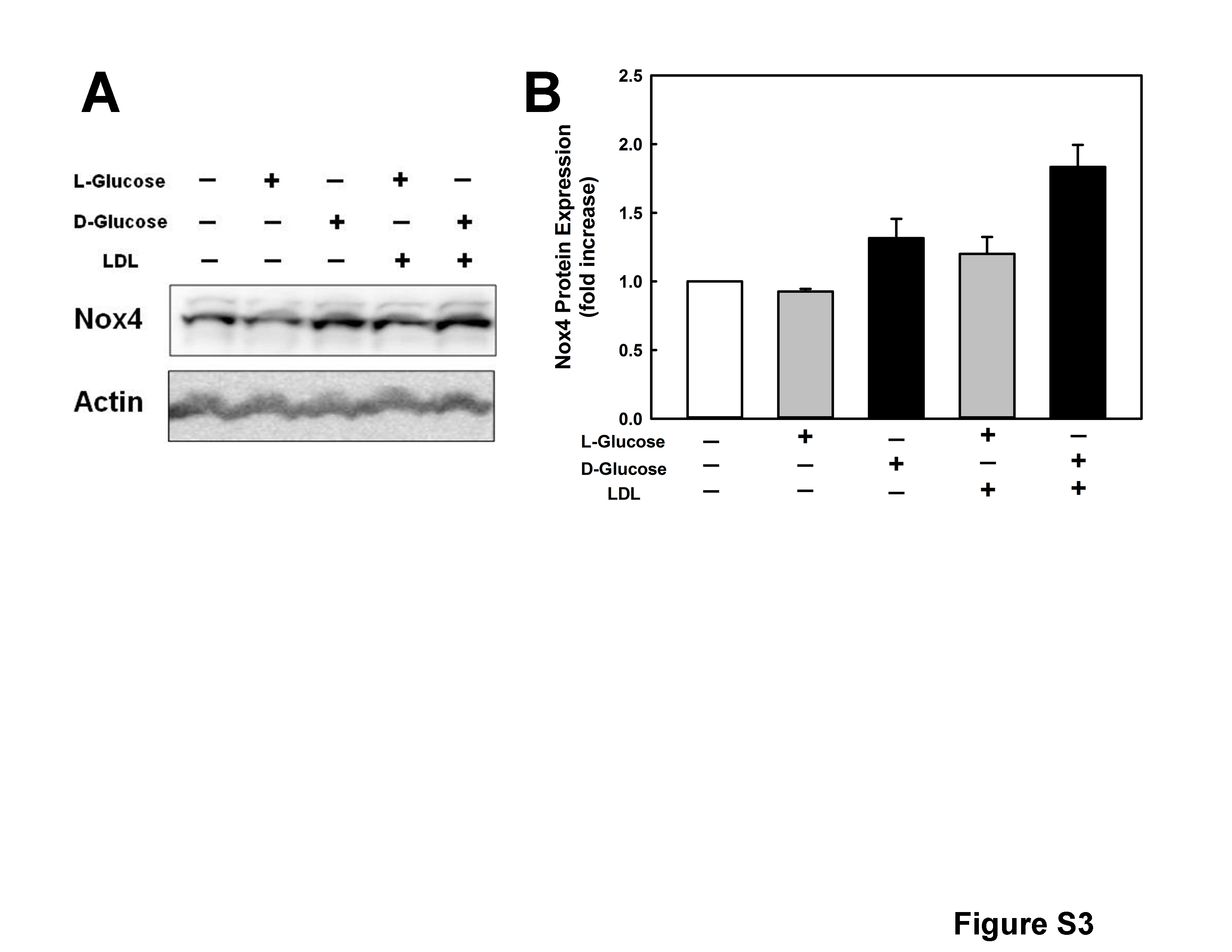

Supplement: Figure S3 — Only D-glucose, not L-glucose, induces Nox4 expression and accelerates monocyte chemotaxis. (A) THP-1 monocytes were preincubated with either 20 mM L-glucose plus 5 mM D-glucose or 25 mM D-glucose where indicated and Nox4 expression was determined. Representative Western blots are shown. (B) Quantification of Nox4 expression levels. n = 2. (TIF) [file pone.0066964.s003.tif]

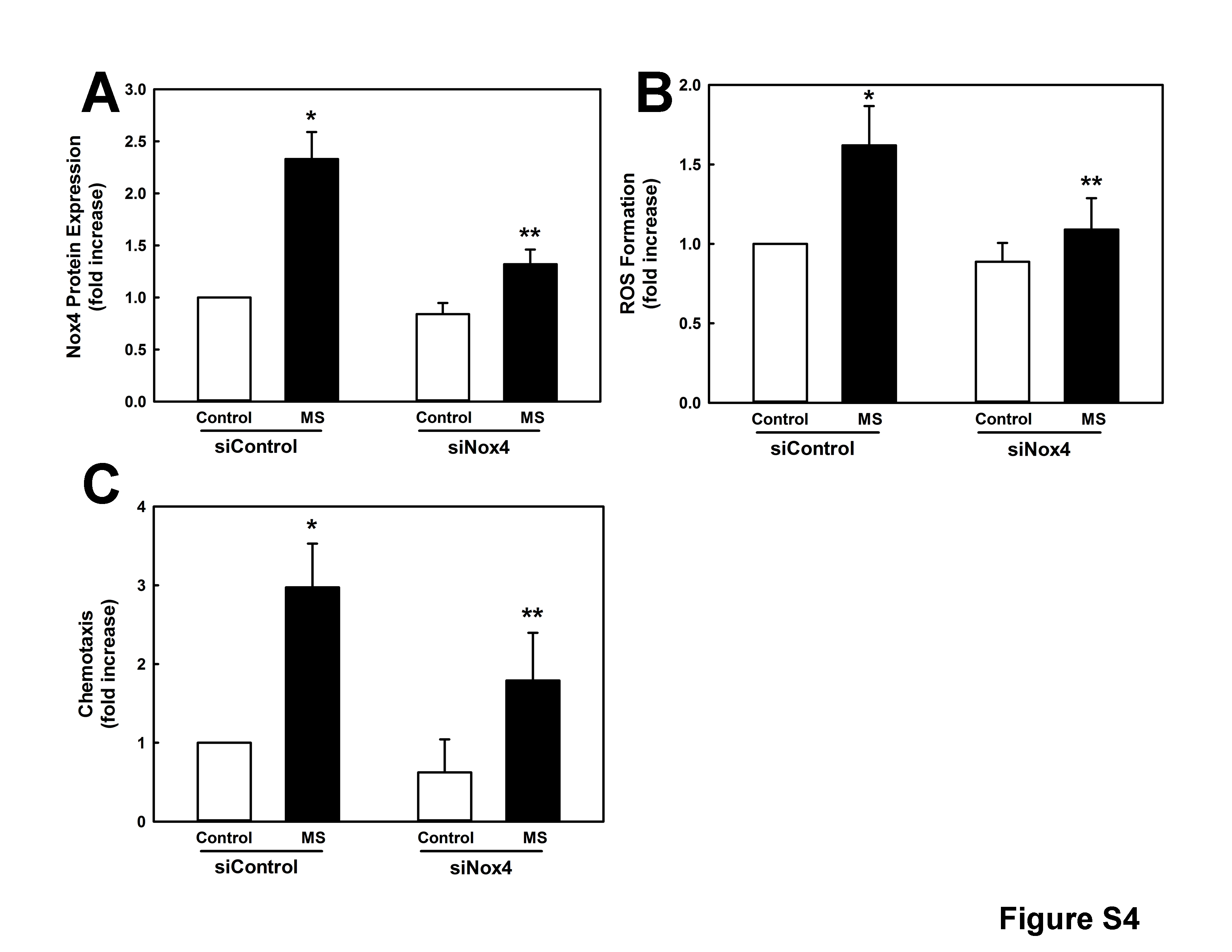

Supplement: Figure S4 — Knockdown of Nox4 suppressed metabolic stress-induced ROS formation and accelerated chemotaxis. THP-1 monocytes were transfected with control or Nox4 siRNAs and then stimulated with control or metabolic stress medium. (A) Nox4 protein expression, (B) ROS formation and (C) MCP-1-induced chemotaxis were measured as described under “Material and Methods.” **: P<0.05 vs MS/siControl. *: P<0.05 vs C/siControl; n = 4. (TIF) [file pone.0066964.s004.tif]

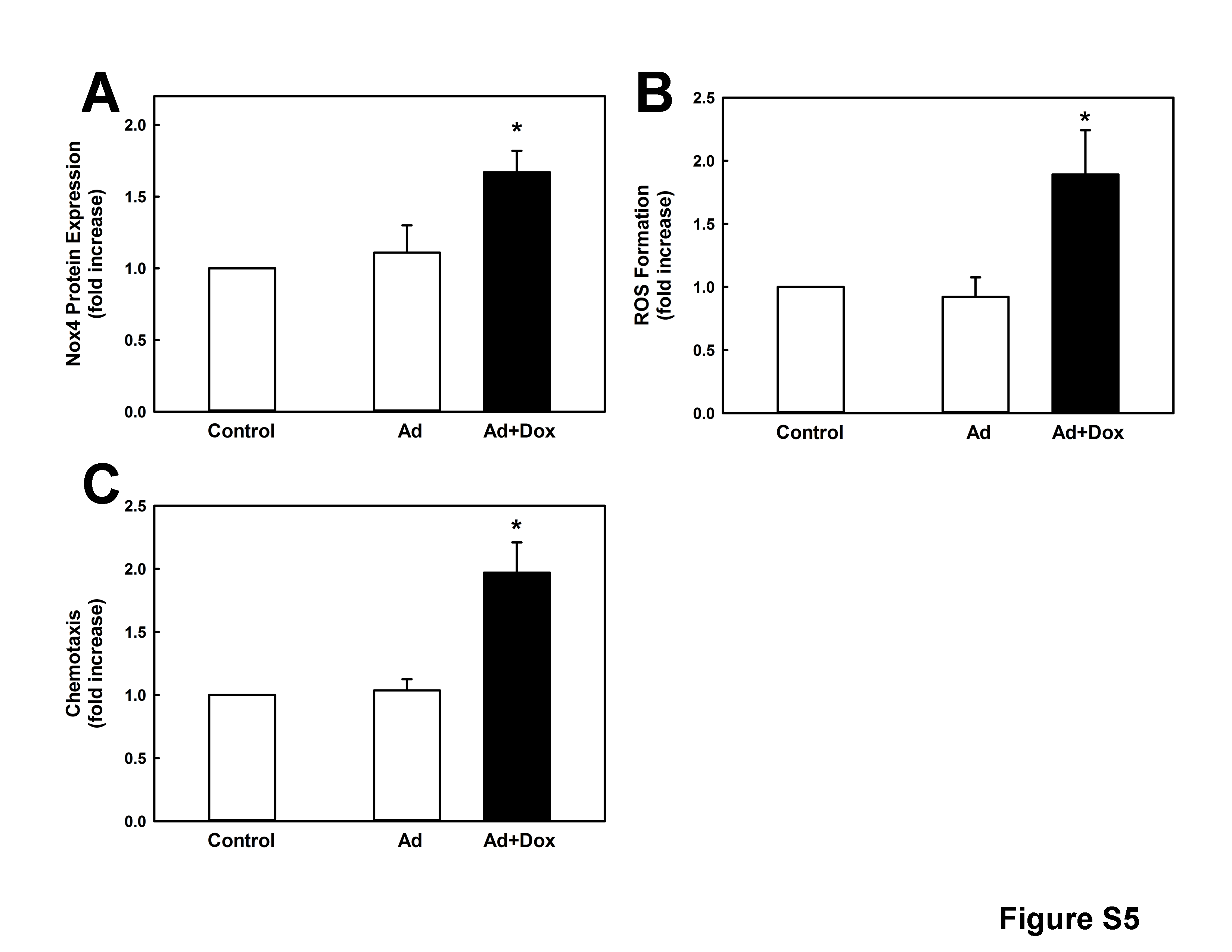

Supplement: Figure S5 — Overexpression of Nox4 promotes OS production and accelerates monocyte chemotaxis. (A) Nox4 was overexpressed in THP-1 monocytes using doxycycline-inducible adenoviruses (MOI = 50; 1 μg/ml Dox). (B) ROS production and (C) chemotaxis was measured as described under “Material and Methods”. *:P<0.05 versus Control; n = 4. (TIF) [file pone.0066964.s005.tif]
